# Preoperative Chemoradiotherapy versus Chemotherapy for Locally Advanced Gastric Cancer or Gastroesophageal Junction Adenocarcinoma: A Phase III Randomized Controlled Trial from China

**DOI:** 10.34133/cancomm.0006

**Published:** 2026-01-27

**Authors:** Xiaowen Liu, Jiejie Jin, Menglong Zhou, Ye Zhou, Hong Cai, Hua Huang, Min Yan, Zhongyin Yang, Runhua Feng, Qi Lu, Hao Ding, Hongtao Xu, Xuexiao Liu, Guichao Li, Hui Zhu, Weiqi Sheng, Xiujiang Yang, Zhen Zhang, Yanong Wang

**Affiliations:** ^1^ Department of Gastric Surgery, Fudan University Shanghai Cancer Center, Shanghai, P. R. China.; ^2^ Department of Oncology, Shanghai Medical College of Fudan University, Shanghai, P. R. China.; ^3^ Department of Radiation Oncology, Fudan University Shanghai Cancer Center, Shanghai, P. R. China.; ^4^ Shanghai Key Laboratory of Radiation Oncology, Fudan University, Shanghai, P. R. China.; ^5^ Department of Gastrointestinal Surgery, Shanghai Institute of Digestive Surgery, Shanghai Key Laboratory of Stomach Neoplasm, Ruijin Hospital, Shanghai Jiaotong University School of Medicine, Shanghai, P. R. China.; ^6^ Department of Surgery, Huadong Hospital, Fudan University, Shanghai, P. R. China.; ^7^ Department of Surgery, Zhejiang Lishui Central Hospital, Lishui, Zhejiang, P. R. China.; ^8^ Department of Radiotherapy, Zhejiang Lishui Central Hospital, Lishui, Zhejiang, P. R. China.; ^9^ Department of Radiology, Fudan University Shanghai Cancer Center, Shanghai, P. R. China.; ^10^ Department of Pathology, Fudan University Shanghai Cancer Center, Shanghai, P. R. China.; ^11^ Institute of Pathology, Fudan University, Shanghai, P. R. China.; ^12^ Department of Endoscopy, Fudan University Shanghai Cancer Center, Shanghai, P. R. China.

## Abstract

**Background:** The prognostic superiority of preoperative chemoradiotherapy (pre-CRT) over preoperative chemotherapy (pre-CT) in patients with locally advanced gastric cancer remains controversial. Herein, we evaluated the efficacy and safety of pre-CRT relative to those of pre-CT in this cohort. **Methods:** This open-label, phase III, randomized controlled trial was conducted at 4 medical centers in China. Eligible patients with locally advanced gastric cancer or esophagogastric junction adenocarcinoma were randomly assigned (1:1) to receive either 3 cycles of oxaliplatin and S-1 (SOX), followed by surgery and 3 postoperative cycles of SOX (pre-CT), or 1 cycle of SOX, followed by concurrent chemoradiotherapy, a second cycle of SOX, surgery, and 3 postoperative cycles of SOX (pre-CRT). The primary endpoint was 3-year disease-free survival (DFS). Secondary endpoints included 3-year overall survival (OS), R0 resection rate, pathological complete response (pCR) rate, treatment-related toxicity, and postoperative complications. **Results:** Due to premature trial termination, only 204 patients were enrolled, and an efficacy analysis was conducted on 194 eligible patients. The baseline characteristics were well balanced between the 2 groups. The DFS and OS were indistinguishable between the 2 groups. The 3-year DFS rates were 53.6% in the pre-CRT group and 53.9% in the pre-CT group [hazard ratio (HR), 1.02; 95% confidence interval (CI), 0.70 to 1.50; log-rank *P* = 0.913]. The 3-year OS rates were 62.8% in the pre-CRT group and 60.5% in the pre-CT group (HR, 0.97; 95% CI, 0.63 to 1.47; log-rank *P* = 0.874). The R0 resection rates were 81.0% and 74.5% in the pre-CRT and pre-CT groups, respectively. Additionally, the pCR rate was higher in the pre-CRT group (12.0%) than in the pre-CT group (2.1%). Treatment-related toxic effects were comparable between the 2 groups. **Conclusion:** This trial did not demonstrate a survival advantage for pre-CRT over pre-CT in patients with locally advanced gastric or gastroesophageal adenocarcinoma.

## Background

Gastric cancer is the fifth most common malignancy and the third leading cause of cancer-related deaths worldwide, and it is especially prevalent in China [1,2]. Its onset and progression are often insidious, with more than two-thirds of patients diagnosed at an advanced stage, resulting in a relatively poor prognosis [3–5]. Multidisciplinary treatment is crucial to improve the prognosis of patients with advanced disease.

Perioperative chemotherapy was first reported to be effective for locally advanced gastric cancer in the MAGIC trial [6]. In the subsequent years, the effects of various chemotherapy regimens have been investigated and compared. In the FLOT4-AIO study [7], the fluorouracil, leucovorin, oxaliplatin, and docetaxel (FLOT) regimen was more effective than the epirubicin, cisplatin, and fluorouracil (ECF) regimen for patients with resectable locally advanced gastric cancer or gastroesophageal junction adenocarcinoma. In the PRODIGY study [8,9], perioperative chemotherapy with docetaxel, oxaliplatin, and S-1 (DOS) regimen improved 3-year disease-free survival (DFS) for the same population. Similarly, in the RESOVLE study [10,11], an oxaliplatin and S-1 (SOX) regimen was effective as perioperative chemotherapy, leading to its increased use in China.

In several phase II studies [12–14], preoperative chemoradiotherapy (pre-CRT) yielded high rates of R0 resection and pathological complete response (pCR). However, whether pre-CRT could be superior to preoperative chemotherapy (pre-CT) was undetermined when the PREACT trial was designed in 2016 [15]. Prior to the PREACT trial, we conducted a prospective, single-arm phase II clinical study [16] to explore the safety and efficacy of pre-CRT in patients with locally advanced gastric cancer, which yielded satisfactory tumor regression and overall survival (OS). Based on those findings, we designed this multicenter, randomized controlled clinical trial [15] to investigate whether pre-CRT offers better survival benefits than pre-CT in patients with locally advanced gastric cancer.

## Methods

### Study design

**Fig. 1.**
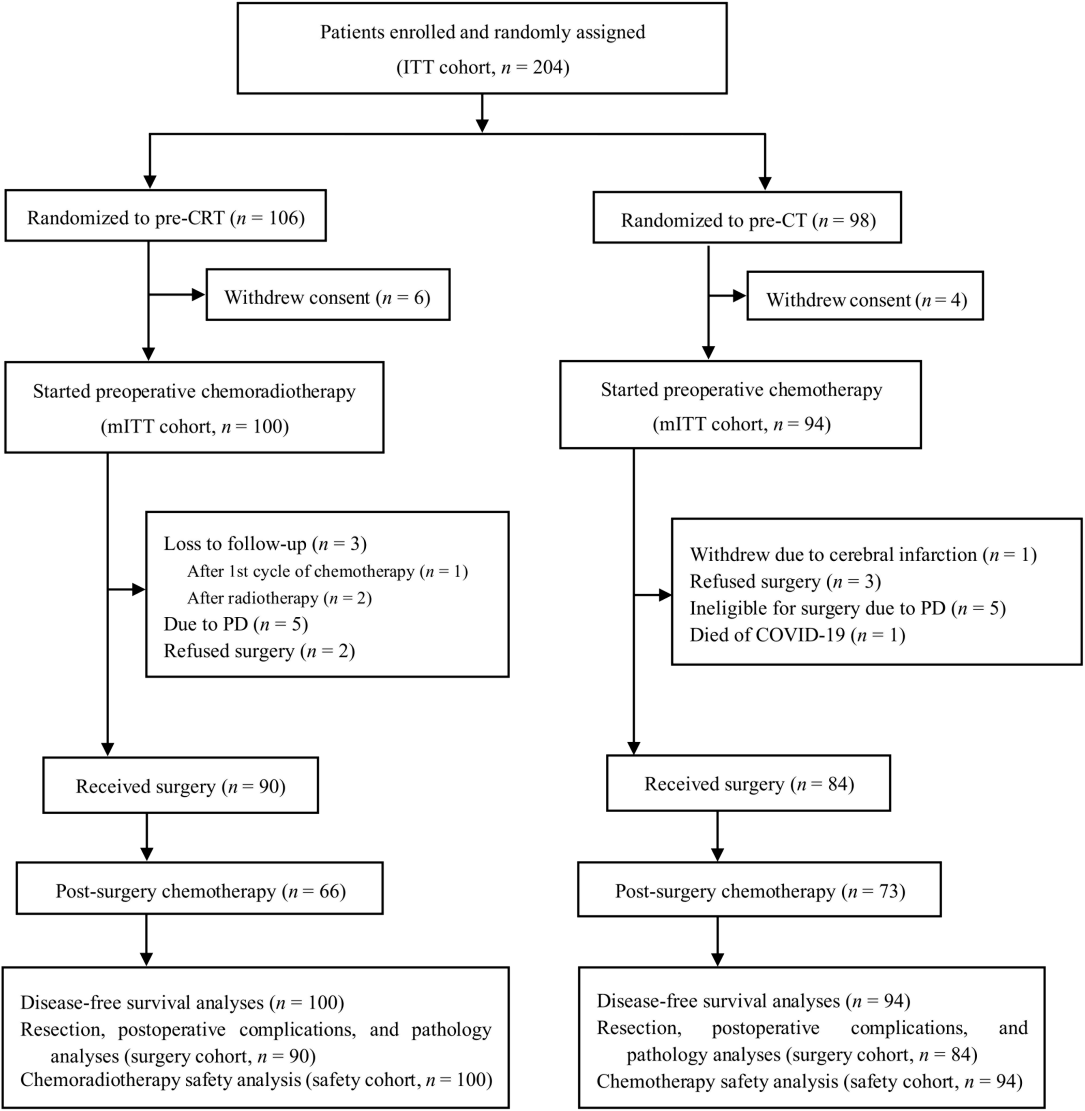
Study profile. ITT, intention-to-treat; mITT, modified intention-to-treat; pre-CRT, preoperative chemoradiotherapy; pre-CT, preoperative chemotherapy; C1, first cycle; SAE, severe adverse event; PD, progressive disease; COVID-19, coronavirus disease 2019.

PREACT is a phase III randomized controlled trial conducted at 4 centers in China, with Fudan University Shanghai Cancer Center serving as the lead institution. The other participating centers include Ruijin Hospital Affiliated to Shanghai Jiao Tong University School of Medicine, Huadong Hospital Affiliated to Fudan University, and Lishui Central Hospital (The Fifth Hospital Affiliated to Wenzhou Medical University). Eligible patients diagnosed with locally advanced gastric cancer or esophagogastric junction adenocarcinoma were randomly assigned to the pre-CRT group or the pre-CT group, both of whom subsequently underwent surgery and postoperative chemotherapy (Fig. 1). Key exclusion criteria were as follows: presence of linitis plastica, evidence of distant metastasis, failure to rule out peritoneal dissemination via staging laparoscopy or laparotomy, and a history of prior chemotherapy or radiotherapy.

In the pre-CRT arm, patients received one cycle of SOX chemotherapy, followed by 45 Gy of radiotherapy administered in 25 fractions (5 fractions per week for 5 consecutive weeks) with concurrent S-1 administration on days 1 to 5 of each treatment week, and subsequently underwent a second cycle of SOX chemotherapy, surgery, and an additional 3 cycles of SOX chemotherapy. In the pre-CT arm, patients completed 3 cycles of SOX before surgery and received 3 cycles of SOX after surgery. The study protocol was approved by the Ethics Committee of Fudan University Shanghai Cancer Center (Approval No. 1611166-2) and the institutional review boards (IRBs) of all other participating centers: Huadong Hospital Affiliated to Fudan University (No. 20170104), Ruijin Hospital Affiliated to Shanghai Jiao Tong University School of Medicine (2018 Clinical Ethics Review No. 116), and Lishui Central Hospital (2018 Clinical Ethics Review No. 56). All participants provided written informed consent before enrollment. The study design was previously described in detail in our full trial protocol [15].

Participants who were randomly assigned but did not receive any chemotherapy were excluded from all analyses. The modified intention-to-treat (mITT) cohort included only patients who received any study treatment. The primary objective of the trial was to investigate whether pre-CRT could improve 3-year DFS compared with pre-CT. Secondary endpoints included the 3-year OS, curative gastrectomy rate, pathological response rate, treatment toxicity, and postoperative complications.

### Participants

Eligible patients were those with histologically confirmed adenocarcinoma of the stomach or gastroesophageal junction (Siewert types II and III) of stage III or IVA (i.e., T3-T4aN+ or T4bN−/+) and were considered operable following initial staging investigations. Additional eligibility criteria included Eastern Cooperative Oncology Group performance status of 0 to 1, adequate bone marrow, liver, and kidney function.

### Randomization and masking

Patients were centrally randomized into the pre-CRT group or the pre-CT group. The randomization procedure was conducted at a 1:1 allocation ratio by using a centralized web-based system, with stratification by the Lauren classification. The allocation sequence was concealed from both investigators and participants until the time of enrollment.

### Procedures

The pre- and postoperative chemotherapy regimens comprised intravenous administration of oxaliplatin at a dosage of 130 mg/m^2^ on day 1, and oral administration of S-1 at a dosage of 40 to 60 mg twice daily on days 1 to 14, followed by a 7-d rest period. The dosage of S-1 was adjusted according to the patient’s body surface area (BSA): Those with a BSA less than 1.25 m^2^ received 80 mg daily; those with a BSA of 1.25 m^2^ or more but less than 1.5 m^2^ received 100 mg daily; and those with a BSA of 1.5 m^2^ or more received 120 mg daily. Concurrent chemoradiotherapy, initiated 1 week after the completion of the first cycle of pre-operative SOX, consisted of 45 Gy in 25 fractions (5 d per week for 5 weeks), administered along with S-1 at a dosage of 40 to 60 mg twice daily on days 1 to 5 of each week of radiotherapy. Intensity-modulated radiation therapy was planned using computed tomography images, with the radiation field encompassing the primary lesion and regional lymph node (LN) drainage.

Patients underwent surgery 3 to 5 weeks after completion of pre-CT, preferably using the D2 gastrectomy approach. The type of gastrectomy performed depended on the location and extent of the primary lesion. For middle-third tumors, a gastric margin of more than 5 cm was recommended, and total gastrectomy was performed. For lower-third tumors, a 2-cm duodenal margin was recommended, and distal subtotal gastrectomy was considered. For upper-third tumors, a 3-cm esophageal margin was recommended, and total gastrectomy was performed. Patients who required distal gastrectomy underwent either a Billroth II or a Roux-en-Y gastrojejunostomy, while those who required total gastrectomy underwent a Roux-en-Y esophagojejunostomy.

In the pre-CRT group, tumor response was evaluated after the second cycle of preoperative SOX using abdominal computed tomography. In the pre-CT arm, this was performed after the third preoperative SOX cycle. All evaluations were conducted according to the Response Evaluation Criteria in Solid Tumors (RECIST), version 1.1. All patients underwent reviews on the first day of each chemotherapy cycle and weekly during chemoradiotherapy. These included physical examination, assessment of toxicity and performance status, full blood counts, and serum biochemistry. Acute toxicities were graded using the National Cancer Institute Common Terminology Criteria for Adverse Events, version 5.0.

### Follow-up

Follow-ups were scheduled at 3-month intervals for at least 2 years, followed by 6-month intervals until 5 years, and at 1-year intervals beyond 5 years. Each visit included a physical examination, serum tumor marker examination, and chest radiography. Abdominal ultrasound and computed tomography were performed alternately every 6 months, with computed tomography scheduled for the first follow-up visit. Endoscopic examinations were conducted annually.

### Statistical analysis

A 10% higher 3-year DFS rate was assumed in favor of the pre-CRT group, and we anticipated a 10% dropout rate. Ultimately, a total of 682 patients were required, with a 5% 2-sided type I error and 80% statistical power. The expected hazard ratio (HR) for the DFS hypotheses was 0.83. Fisher’s exact test was used to compare categorical patients’ characteristics between the pre-CRT and pre-CT groups, while Student’s *t* test was used for the analysis of continuous variables. Time-to-event curves for DFS and OS were calculated with the Kaplan–Meier method. Secondary endpoints, including R0 resection rate, pathological response rate, treatment toxicity, and postoperative complications, were initially planned to be compared using the chi-square test; however, due to the early termination of this clinical study, Fisher’s exact test was ultimately adopted instead. Missing data for baseline and intraoperative variables were not imputed. A post hoc sensitivity analysis was conducted for the primary endpoint in patients who underwent R0 resection. All statistical tests were 2-sided, and the level of significance was set to *P* < 0.05.

## Results

### Baseline characteristics

From December 2016 to June 2022, 204 patients were enrolled in the trial across 4 centers in China. Slow participant enrollment led to the premature termination of the trial. Of these, 106 (52.0%) and 98 (48.0%) patients were assigned to the pre-CRT and pre-CT groups, respectively. Ten patients (4.9%) withdrew without receiving any chemotherapy or surgery and were therefore excluded from all analyses. The mITT cohort included only those who were randomly assigned and received any study treatment. In total, 194 patients (95.1%) were included in the mITT cohort [100 (51.5%) in the pre-CRT group and 94 (48.5%) in the pre-CT group]. The surgery cohort comprised 174 patients who underwent surgical interventions [90 (51.7%) in the pre-CRT group and 84 (48.3%) in the pre-CT group]. The safety cohort comprised participants who received at least one cycle of chemotherapy (same as the mITT cohort).

No statistical differences in the baseline characteristics were observed between the 2 groups in the mITT cohort (Table 1). In the pre-CRT group, 21.0% of patients were female, compared to 14.9% in the pre-CT group (*P* = 0.351). Tumors were located at the gastroesophageal junction in 49.0% of patients in the pre-CRT group and 52.1% of patients in the pre-CT group, while 31.0% and 28.7% of patients in the respective groups had tumors in the lower third of the stomach (*P* = 0.910). All tumors were staged as clinical T3/T4 (cT3/T4) at diagnosis. In the pre-CRT group, 65.0% of tumors were classified as clinical T4a (cT4a) and 4.0% as clinical T4b (cT4b), whereas in the pre-CT group, 66.0% were classified as cT4a and 5.3% as cT4b (*P* = 0.875). In the pre-CRT group, 98.0% of patients had LN involvement, and only 2 patients (2.0%) were LN-negative, both of whom had cT4b tumors.

**Table 1.** Baseline characteristics of the mITT cohort. The mITT cohort included patients who were randomly assigned and received any study treatment.

Characteristics	Treatment group		*P* values
	Pre-CRT (*n* = 100)	Pre-CT (*n* = 94)	
Sex, *n* (%)			0.351
Male	79 (79.0%)	80 (85.1%)	
Female	21 (21.0%)	14 (14.9%)	
Age, years (mean ± SD)	61.8 ± 9.4	61.0 ± 10.0	0.557
Tumor location, *n* (%)			0.910
Gastroesophageal junction	49 (49.0%)	49 (52.1%)	
Upper or middle third	20 (20.0%)	18 (19.2%)	
Lower third	31 (31.0%)	27 (28.7%)	
cT stage, *n* (%)			0.875
cT3	31 (31.0%)	27 (28.7%)	
cT4a	65 (65.0%)	62 (66.0%)	
cT4b	4 (4.0%)	5 (5.3%)	
cN stage, *n* (%)			0.745
cN0	2 (2.0%)	0 (0.0%)	
cN1	23 (23.0%)	25 (26.6%)	
cN2	55 (55.0%)	57 (60.6%)	
cN3	20 (20.0%)	12 (12.8%)	
Lauren classification, *n* (%)			0.687
Intestinal type	32 (32.0%)	33 (35.1%)	
Diffuse type	29 (29.0%)	20 (21.3%)	
Mixed type	25 (25.0%)	26 (27.7%)	
Uncertain	14 (14.0%)	15 (16.0%)	
Hemoglobin level, g/l (mean ± SD)	127.0 ± 20.9	124.9 ± 21.9	0.505

Pre-CRT, preoperative chemoradiotherapy; Pre-CT, preoperative chemotherapy; mITT, modified intention-to-treat; cT stage, clinical T stage; cN stage, clinical N stage

### Treatment compliance

In the pre-CT group, 93 patients (98.9%) completed one cycle of preoperative SOX, 91 patients (96.8%) completed 2 cycles, and 86 patients (91.5%) completed all 3 planned cycles (Table 2). In contrast, in the pre-CRT group, 100 patients (100.0%) received the first cycle of chemotherapy, 90 patients (90.0%) completed the concurrent chemoradiotherapy, 78 (78.0%) patients completed the second cycle of chemotherapy, and only 77 (77.0%) patients completed the full pre-CRT regimen. The completion rate in the pre-CRT group was significantly lower than that in the pre-CT group (*P* = 0.006). Following surgery, 48 patients (48.0%) in the pre-CRT group and 52 patients (55.3%) in the pre-CT group completed all 3 planned postoperative SOX cycles (*P* = 0.319).

**Table 2.** Treatment compliance in the mITT cohort. The mITT cohort included patients who were randomly assigned and received any study treatment. Regimen of the pre-CRT arm: 1 cycle SOX → 45 Gy radiotherapy (25 fractions, 5/week) + concurrent S-1 (days 1 to 5 weekly) → 1 cycle SOX → surgery → 3 cycles SOX. Regimen of the pre-CT arm: 3 cycles SOX → surgery → 3 cycles SOX.

Interventions	Treatment group		*P* values
	Pre-CRT (*n* = 100)	Pre-CT (*n* = 94)	
Concurrent chemoradiotherapy, *n* (%)			NA
Received radiation	92 (92.0%)	NA	
Received 45 Gy	90 (90.0%) ^a^	NA	
Preoperative chemotherapy, *n* (%)			0.010
Received all cycles ^b^	78 (78.0%) ^c^	86 (91.5%)	
Preoperative treatment, *n* (%)			0.006
Received all cycles ^d^	77 (77.0%)	86 (91.5%)	
Postoperative chemotherapy, *n* (%)			0.319
Received all 3 cycles	48 (48.0%)	52 (55.3%)	
Surgery, *n* (%)			>0.999
No surgery	10 (10.0%)	10 (10.6%)	
Received surgery	90 (90.0%)	84 (89.4%)	
Median interval to surgery, weeks	4.9	4.5	0.406

NA, not applicable

^a^ Two patients did not complete the full 45-Gy radiotherapy course. One withdrew from the study due to disease progression during radiotherapy, while the other discontinued radiotherapy because of intolerable radiation esophagitis.

^b^ Pre-CRT arm: 2 cycles SOX; pre-CT arm: 3 cycles SOX.

^c^ One patient in the pre-CRT group discontinued radiotherapy after the 20th fraction for intolerable radiation esophagitis but received the second cycle of SOX.

^d^ Pre-CRT arm: 2 cycles SOX + 45 Gy radiotherapy; pre-CT arm: 3 cycles SOX.

The proportion of patients who underwent surgery was 90.0% (90/100) and 89.4% (84/94) in the pre-CRT and pre-CT groups, respectively (Fig. 1). Ten patients (10.6%) in the pre-CT group did not undergo surgery: 1 (1.1%) owing to cerebral infarction, 3 (3.2%) who refused surgery and opted for continued chemotherapy, 5 (5.3%) owing to disease progression, and 1 (1.1%) who died from coronavirus disease 2019 (COVID-19) during preoperative treatment. In the pre-CRT group, 10 (10.0%) patients also did not undergo surgery: 5 (5.0%) owing to disease progression, 2 (2.0%) who refused surgery, and 3 (3.0%) who were lost to follow-up during preoperative treatment.

### Efficacy

The median interval from the completion of preoperative therapy to surgery was 4.9 weeks in the pre-CRT group and 4.5 weeks in the pre-CT group (Table 2). Additional post hoc analyses of surgical and pathological outcomes in the surgery cohort were reported in Table 3. In the pre-CRT group, 67 patients (67.0%) underwent total gastrectomy, and 17 patients (17.0%) underwent distal gastrectomy. In the pre-CT group, 65 patients (69.1%) underwent total gastrectomy and 13 (13.8%) underwent distal gastrectomy. The R0 resection rates were 81.0% (81/100) in the pre-CRT group and 74.5% (70/94) in the pre-CT group (*P* = 0.052; Table 3). Additionally, 83.0% of the patients in each group underwent D2 LN dissection (Table 3). The proportion of patients diagnosed with ypT0-T1 was 25.0% in the pre-CRT group, higher than the 11.7% in the pre-CT group, but the difference was not statistically significant (*P* = 0.339). Meanwhile, 42.0% of patients in the pre-CRT group were diagnosed with ypN0, which was significantly higher than the 21.3% observed in the pre-CT group (*P* = 0.005). Notably, the pCR (defined as pT0N0) rate was 12.0% (12/100) in the pre-CRT group, which was significantly higher than that in the pre-CT group [2.1% (2/94); *P* = 0.011].

**Table 3.** Surgical and pathological outcomes in the mITT cohort. The mITT cohort included patients who were randomly assigned and received any study treatment.

Variables	Pre-CRT (*n* = 100)	Pre-CT (*n* = 94)	*P* values
Curative intent surgery, *n* (%)	90 (90.0%)	84 (89.4%)	>0.999
Surgical extent, *n* (%)			1.000
Total gastrectomy	67 (67.0%)	65 (69.1%)	
Distal gastrectomy	17 (17.0%)	13 (13.8%)	
Laparotomy/gastrojejunostomy	6 (6.0%)	6 (6.4%)	
Resection margin ^a^, *n* (%)			0.052
R0	81 (81.0%)	70 (74.5%)	
R1	0 (0.0%)	7 (7.4%)	
R2	3 (3.0%)	1 (1.1%)	
Lymphadenectomy, *n* (%)			1.000
D2	83 (83.0%)	78 (83.0%)	
Others	7 (7.0%) ^b^	6 (6.4%)	
Tumor stage ^a^, *n* (%)			0.339
ypT0 ^c^	13 (13.0%)	5 (5.3%)	
ypT1	12 (12.0%)	6 (6.4%)	
ypT2	11 (11.0%)	11 (11.7%)	
ypT3	33 (33.0%)	43 (45.7%)	
ypT4	15 (15.0%)	13 (13.8%)	
Nodal stage ^a^, *n* (%)			0.005
ypN0	42 (42.0%)	20 (21.3%)	
ypN1	18 (18.0%)	31 (33.0%)	
ypN2	12 (12.0%)	12 (12.8%)	
ypN3	12 (12.0%)	15 (16.0%)	
pCR rate, *n* (%)	12 (12.0%)	2 (2.1%)	0.011

pCR, pathological complete response

^a^ Resection margin status, tumor stage, and nodal stage were evaluated only in patients who underwent tumor resection (pre-CRT, *n* = 84; pre-CT, *n* = 78).

^b^ One patient underwent D1 lymphadenectomy, while 6 patients received laparotomy/gastrojejunostomy.

^c^ The yp prefix represents cases in which pathological staging was performed following preoperative therapy.

**Fig. 2.**
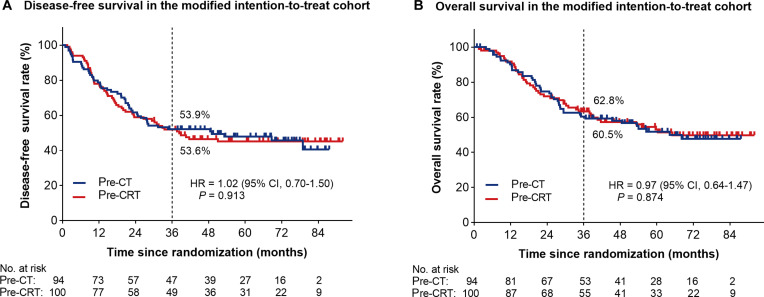
Kaplan–Meier estimates of survival in the modified intention-to-treat cohort. (A) The disease-free survival curves. (B) The overall survival curves. Pre-CT, preoperative chemotherapy; Pre-CRT, preoperative chemoradiotherapy; HR, hazard ratio; 95% CI, 95% confidence interval; No., number.

**Fig. 3.**
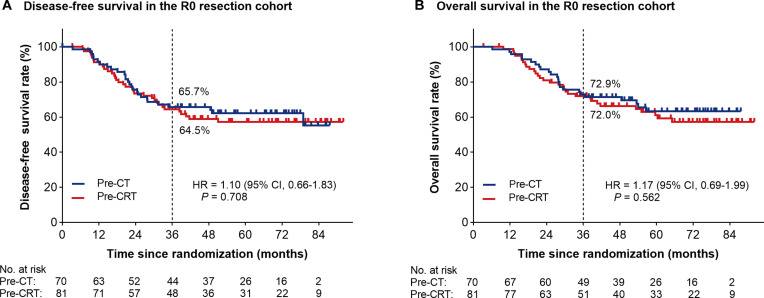
Kaplan–Meier estimates of survival in the R0 resection population. (A) The disease-free survival curves. (B) The overall survival curves. Pre-CT, preoperative chemotherapy; Pre-CRT, preoperative chemoradiotherapy; HR, hazard ratio; 95% CI, 95% confidence interval; No., number.

The median follow-up time was 42.4 months. During the 3-year follow-up period, 46 patients (46.0%) in the pre-CRT group died, including 43 (93.5%) from gastric cancer and 3 (6.5%) from other causes, whereas 44 patients (46.8%) in the pre-CT group died, including 42 (95.5%) from gastric cancer and 2 (4.5%) from other causes. In terms of DFS, 98 events were recorded in the mITT population, including 51 patients (51.0%) in the pre-CRT group and 47 patients (50.0%) in the pre-CT group. Until the data censoring on 2024 December 5, the estimated 3-year DFS rates in the mITT cohort were 53.6% in the pre-CRT group and 53.9% in the pre-CT group. The HR for DFS events in the pre-CRT group versus those in the pre-CT group was 1.02 [95% confidence interval (CI), 0.70 to 1.50; log-rank *P* = 0.913; Fig. 2A]. Regarding OS analysis, 90 events were recorded in the mITT population, including 46 events (46.0%) among 100 patients in the pre-CRT group, and 44 events (46.8%) among 94 patients in the pre-CT group. The estimated 3-year OS rates in the mITT cohort were 62.8% in the pre-CRT group and 60.5% in the pre-CT group (HR, 0.97; 95% CI, 0.64 to 1.47; log-rank *P* = 0.874; Fig. 2B). Post hoc sensitivity analysis was conducted in the R0 resection cohort. The estimated 3-year DFS rates were 64.5% in the pre-CRT group and 65.7% in the pre-CT group (HR, 1.10; 95% CI, 0.66 to 1.83; *P* = 0.708; Fig. 3A). The estimated 3-year OS rates were 72.0% in the pre-CRT group and 72.9% in the pre-CT group (HR, 1.17; 95% CI, 0.69 to 1.99; *P* = 0.562; Fig. 3B).

### Safety

Adverse events of any grade were recorded in 79 patients (79.0%) in the pre-CRT group and 76 (80.9%) in the pre-CT group. Gastrointestinal and hematologic toxicities were the main acute toxic effects (grade 3 or higher) associated with perioperative treatment, as shown in Table 4. No substantial difference was observed in overall gastrointestinal toxicity between the 2 groups. In the pre-CRT group, 14.0% of patients experienced major gastrointestinal toxicity, whereas 10.6% did in the pre-CT group (*P* = 0.519). Similarly, no substantial difference was observed in overall hematologic cytotoxicity between the 2 groups. In the pre-CRT group, 31.0% of patients experienced major hematologic toxicity, compared to 28.7% in the pre-CT group (*P* = 0.756). However, the groups differed in terms of the incidence of severe anemia (Fisher’s exact *P* = 0.029), with 2.0% of patients in the pre-CRT group and 9.6% of patients in the pre-CT group. Severe thrombocytopenia was observed in 20 (20.0%) and 13 (13.8%) patients in the pre-CRT and pre-CT groups, respectively; however, the difference was not significant (*P* = 0.339). Similarly, severe neutropenia was comparable between groups, occurring in 8 patients (8.0%) in the pre-CRT group and in 4 patients (4.3%) in the pre-CT group (*P* = 0.375).

**Table 4.** Gastrointestinal and hematological toxicities before and after surgery in the safety cohort. The safety cohort consisted of participants who received at least one cycle of chemotherapy.

Toxicity grade 3 or higher	Treatment group		*P* values
	Pre-CRT (*n* = 100)	Pre-CT (*n* = 94)	
Gastrointestinal toxicities, *n* (%)			
Nausea	2 (2.0%)	1 (1.1%)	>0.999
Vomiting	4 (4.0%)	3 (3.2%)	>0.999
Dysphagia	2 (2.0%)	0 (0.0%)	0.498
Esophagitis	2 (2.0%)	0 (0.0%)	0.498
Anorexia	4 (4.0%)	2 (2.1%)	0.684
Diarrhea	0 (0.0%)	2 (2.1%)	0.234
Hematemesis	2 (2.0%)	2 (2.1%)	>0.999
Total	14 (14.0%)	10 (10.6%)	0.519
Hematological toxicities, *n* (%)			
Neutropenia	8 (8.0%)	4 (4.3%)	0.375
Leukopenia	2 (2.0%)	1 (1.1%)	>0.999
Anemia	2 (2.0%)	9 (9.6%)	0.029
Thrombocytopenia	20 (20.0%)	13 (13.8%)	0.339
Total	31 (31.0%)	27 (28.7%)	0.756

Pre-CRT, preoperative chemoradiotherapy; pre-CT, preoperative chemotherapy.

The 2 groups did not appreciably differ in terms of surgical complications. In the pre-CRT group, 15 patients (16.7%) experienced grade 3 or higher surgical complications, compared to 11 (13.1%) in the pre-CT group (*P* = 0.532; Table 5). In the pre-CRT group, 6 patients (6.7%) developed intra-abdominal sepsis, while 3 patients (3.6%) in the pre-CT group did (*P* = 0.499). Anastomotic leakage (including duodenal stump leakage) occurred in 3 patients (3.3%) in the pre-CRT group and in 1 patient (1.2%) in the pre-CT group (*P* = 0.622). Postoperative bleeding occurred in 1 patient (1.1%) in the pre-CRT group and in 3 patients (3.6%) in the pre-CT group (*P* = 0.354). In all cases, the bleeding was cured with conservative treatment. One patient (1.1%) in the pre-CRT group who underwent total gastrectomy experienced an anastomotic stricture, whereas none in the pre-CT group did. Both groups had one case of postoperative gastroparesis, occurring in patients who underwent gastrointestinal bypass surgery.

**Table 5.** Surgical complications in the surgery cohort. The surgery cohort consists of patients who received surgical interventions.

Surgical complications (grade 3 or higher)	Treatment group		*P* values
	Pre-CRT (*n* = 90)	Pre-CT (*n* = 84)	
Intra-abdominal sepsis	6 (6.7%)	3 (3.6%)	0.499
Anastomotic leak	3 (3.3%)	1 (1.2%)	0.622
Bleed	1 (1.1%)	3 (3.6%)	0.354
Anastomotic stricture	1 (1.1%)	0 (0.0%)	>0.999
Wound infection	1 (1.1%)	1 (1.2%)	>0.999
Chest infection	2 (2.2%)	2 (2.4%)	>0.999
Gastroparesis	1 (1.1%)	1 (1.2%)	>0.999
Death	0 (0.0%)	0 (0.0%)	>0.999
Overall surgical complications	15 (16.7%)	11 (13.1%)	0.532

Pre-CRT, preoperative chemoradiotherapy; Pre-CT, preoperative chemotherapy.

## Discussion

Perioperative treatment is the standard treatment mode for patients with locally advanced gastric cancer, and perioperative chemotherapy has been shown to improve the outcomes of such patients [6–11]. Although radiotherapy may play a crucial role in the management of this patient population, evidence regarding its added benefit remains inconsistent, with some studies demonstrating efficacy while others failing to show an improvement in prognosis when radiotherapy is added to postoperative chemotherapy [17–21]. Therefore, whether the addition of radiotherapy to pre-CT improves the prognosis of such patients remains to be determined. At the beginning of PREACT, the results of only one prematurely terminated phase III trial, POET, were published [22,23]. Therefore, we conducted a series of investigations on pre-CRT. We first conducted a phase II clinical trial, which showed that satisfactory pCR and R0 resection rates could be achieved through pre-CRT without obvious surgical complications [16]. Subsequently, we designed this phase III trial to investigate whether pre-CRT could improve the prognosis compared with pre-CT. Because of differences between Eastern and Western patients with gastric cancer, such as the high incidence of peritoneal metastasis and poor tolerance to chemotherapy drugs among the former, we used laparoscopic exploration as the screening method for enrollment, and the 3-drug regimens, such as ECF or FLOT (docetaxel, oxaliplatin, and fluorouracil), were replaced with the SOX regimen (S-1 plus oxaliplatin). Unfortunately, this study was terminated early owing to slow enrollment, which was caused by 2 main factors. First, laparoscopic exploration identified a large number of patients with peritoneal metastases or positive cytology. Second, the COVID-19 pandemic occurred during the enrollment period. Finally, we enrolled only 204 patients in the study, despite the estimated requirement of 682. After more than 3 years of follow-up, our results suggest that pre-CRT did not provide survival benefits compared with pre-CT. These results are consistent with those of the recently published TOPGEAR study [24].

R0 resection and pCR rates were secondary endpoints of this trial. R0 resection is associated with survival [25,26]. A network meta-analysis showed that R0 resection was more frequent after pre-CRT than after pre-CT [27]. In this trial, the R0 resection rate in the pre-CRT group was 81.0%, which was higher than that of the pre-CT group (74.5%); however, this difference was not statistically significant. This result is the same as that of the TOPGEAR study and the POET study [22–24]. Meanwhile, we noted that the R0 resection rate in the pre-CRT group varied across these 3 trials. The R0 resection rate in the pre-CRT group was lower than that in the TOPGEAR study (92.0%), but higher than that in the POET study (72.0%) [22–24]. These differences may in part be attributable to the different clinical stages of the enrolled patients. All patients in the pre-CRT group had stage T3 or T4 disease (stage T4, 69.0%), similar to those in the POET study [22,23]. However, 12.0% of patients in the pre-CRT group had stage T1 or T2 disease in the TOPGEAR study [24,28]. Another surrogate endpoint in neoadjuvant therapy is pCR. In this study, the pCR rates in the pre-CRT and pre-CT groups were notably different, at 12.0% and 2.1%, respectively. These results demonstrate that pre-CRT has obvious advantages over pre-CT in terms of tumor regression. These pCR rates are similar to those in POET (15.6% versus 2.0%) and slightly lower than those in TOPGEAR (17.0% versus 8.0%). The differences in the pCR rates may be related to the different tumor burdens of the enrolled patients. Ninety-eight percent of patients were diagnosed with LN metastasis in this study, whereas in the TOPGEAR study, less than 70.0% of patients had LN metastasis. Studies have demonstrated a strong correlation between the pCR rate and the prognosis of gastric cancer [29,30]. Although the pCR rate was pronouncedly higher in the pre-CRT group, it did not translate into a better prognosis. This dilemma is also seen with preoperative immunotherapy, as in the KEYNOTE-585 study [31]. This phenomenon may be explained by the fact that parameters such as pCR and R0 resection rates reflect local treatment effects, while locally advanced gastric cancer remains a systemic disease, so improved local control does not necessarily prevent distant recurrence. Therefore, new systemic treatment options, such as immunotherapy and targeted therapy, should be investigated for patients with locally advanced gastric cancer.

Treatment completion is related to the treatment effect. Chemoradiotherapy before or after surgery has a considerable impact on the treatment completion [21,28]. In the CRITICS study, only 62.0% of patients received chemoradiotherapy postoperatively [21]. In the present study, 77% of patients in the pre-CRT group completed the pre-CRT regimen. Meanwhile, in the TOPGEAR study, 91% patients in the pre-CRT group received pre-CRT [24]. These differences may be attributable to our study design. In the pre-CRT group, preoperative therapy was administered using a sandwich method: 2 cycles of chemotherapy with a cycle of concurrent chemoradiotherapy in between. Although this protocol could shorten the overall treatment time, it can also shorten the time that the patient has to recover, thus leading to a weakened tolerance for chemotherapy. The safety analysis showed that the incidence of any treatment-related toxicity was 81.0% in the pre-CRT group and 80.9% in the pre-CT group, with no appreciable difference. The adverse events were mainly gastrointestinal toxicity and bone marrow suppression. In the pre-CRT and pre-CT groups, the incidence of grade 3 or higher surgical complications was comparable (16.7% and 13.1%, respectively). Similarly, the incidence of anastomotic leakage was not substantially different between the 2 groups (3.3% and 1.2%, respectively).

Strict quality control measures were applied in this trial. First, to exclude patients with peritoneal metastases, laparoscopic exploration was mandatory for all patients. Secondly, based on phase II results that patients with linitis plastica showed a poor therapeutic response to pre-CRT, such patients were excluded from this study. In order to reduce the influence of breathing movements on the accuracy of radiotherapy, specific methods have been investigated [32,33]. The surgical quality in this trial was consistently high, as all participating surgeons were senior clinicians with extensive operative experience, and surgical techniques were centrally reviewed. Ultimately, 83.0% of patients underwent D2 gastrectomy.

This trial has several notable strengths. First, it was the first phase III clinical trial in Asia to compare pre-CRT to pre-CT. As gastric cancer differs substantially between Eastern and Western populations, owing to variations in ethnicity, chemotherapy regimens, and LN dissection, the results of the TOPGEAR trial are not directly applicable to Asian patients with gastric cancer. Therefore, the PREACT trial offers a crucial perspective that complements the international landscape of gastric cancer research as the Asian counterpart to TOPGEAR. The similar results of these 2 studies indicate that the efficacy of pre-CRT for advanced gastric cancer is yet to be validated globally. Second, laparoscopic exploration was mandatory, and patients with gross and microscopic intra-abdominal metastases were excluded. Laparoscopy ensured that the disease was locally advanced and minimized the risk of inadvertent inclusion of patients with peritoneal disease. Additionally, laparoscopy enabled standardization of D2 resection, which enhanced the credibility of the surgical outcomes compared with Western studies. Third, this trial dispelled the concerns that chemoradiotherapy increases the risk of surgical complications. Finally, the PREACT study can be used as a baseline reference for future clinical research on the efficacy of combined immunotherapy and radiotherapy.

This trial also has several limitations. First, it included both gastric and esophagogastric junction cancers; however, these malignancies exhibit distinct biological characteristics that may influence the effectiveness of chemoradiotherapy. Second, early termination prevented the trial from reaching the planned sample size, resulting in reduced statistical power. Third, the limited number of participating centers may have undermined the generalizability of the results.

Several exploratory directions for future pre-CRT trials may be considered. Immunotherapy, particularly therapies targeting programmed cell death protein 1/programmed death-ligand 1 inhibitors, has been widely adopted for the treatment of patients with advanced gastric cancer [34–37]. For locally advanced gastric cancer, in the MATTERHORN study [38], the addition of immunotherapy to perioperative chemotherapy pronouncedly improved patient survival. Phase II clinical trials [39,40] of pre-CRT combined with immunotherapy have revealed high pCR rates, rendering it a highly promising research direction. The identification of patients most likely to benefit from pre-CRT is also a research priority. For example, the ARTIST study [17] found that chemoradiotherapy may be better for patient groups defined by specific biomarkers. Other potential biomarkers, such as circulating tumor DNA, have the potential to identify patients with lower metastatic potential who might benefit the most from intensified local therapy [41,42].

Overall, although pre-CRT improved the pCR rate compared with pre-CT, it did not result in an improved prognosis. This conclusion is consistent with those of the TOPGEAR and POET trials.

## Ethical Approval

All procedures involving human participants were approved by the Institutional Review Board of Fudan University Shanghai Cancer Center (Approval No. 1611166-2), as well as the ethics committees of the participating centers: Huadong Hospital Affiliated to Fudan University (No. 20170104), Ruijin Hospital Affiliated to Shanghai Jiao Tong University School of Medicine (2018 Clinical Ethics Review No. 116), and Lishui Central Hospital (2018 Clinical Ethics Review No. 56). All experiments were performed in accordance with the principles of the 1964 Declaration of Helsinki and its later amendments or comparable ethical standards. Written informed consent was obtained from all individual participants included in the study.

## Data Availability

The datasets generated and/or analyzed during the current study are available from the corresponding authors on reasonable request.
